# The relationship between executive functions, decision-making, and changes in symptoms of gambling disorder in online sports bettors

**DOI:** 10.1038/s41598-026-48449-8

**Published:** 2026-04-12

**Authors:** Theresa Wirkus, Robert Czernecka, Gerhard Bühringer, Anja Kräplin

**Affiliations:** 1 Faculty of Psychology, Technische Universität Dresden, Dresden, Germany; 2 IFT Mental Health Solutions, Munich, Germany; 3 Department of Psychiatry and Psychotherapy, Technische Universität Dresden, Dresden, Germany

**Keywords:** Gambling disorder, Decision-making, Impulsivity, Loss aversion, Executive functions, Diseases, Health care, Psychology, Psychology, Risk factors

## Abstract

Deficits in executive functions (EFs) and impulsive decision-making are well-established cognitive mechanisms underlying gambling disorder (GD), though there are some inconsistent findings. This study investigated the relationship between changes in GD symptoms in online sports bettors and specific EFs as well as decision-making processes over time. A subsample of online sports bettors (*n* = 54, mean age = 33, 96% male) from a larger study underwent an in-person assessment. Initial GD symptom severity was measured online according to DSM-5 criteria and was low to mild in most participants. All other assessments took place an average of 443 days later, during the in-person assessment. We analyzed cross-sectional and longitudinal associations between three facets of EFs (response inhibition, cognitive flexibility, and working memory) and four decision-making facets (delay discounting, probability discounting for gains and losses, and loss aversion) and GD symptom severity and changes over time. Cross-sectional results showed that higher GD symptom severity was associated with poorer working memory and reduced loss aversion. Reduced loss aversion was also associated with greater symptom increase over time. However, these associations were not statistically significant after correction for multiple testing, so they should be interpreted with caution. No other significant relationships were found. These findings provide preliminary evidence that reduced loss aversion is one cognitive factor in disordered gambling among online sports bettors, a population with heterogeneous profiles of EFs and decision-making. Further longitudinal studies are needed to clarify the role of specific cognitive processes in the development and progression of GD to better inform treatment strategies.

**Supplementary Information:**

The online version contains supplementary material available at 10.1038/s41598-026-48449-8.

## Introduction

Gambling disorder (GD) as an example of addictive disorders is amongst others characterized through deficits in specific executive functions (EFs) and in distinct impulsive decision-making processes. From a theoretical perspective, impairments in EFs and alterations in decision-making are commonly embedded in dual-process models of addiction and self-control. These models distinguish between top-down cognitive control processes, such as response inhibition, working memory, and cognitive flexibility, and bottom-up valuation and reward-processing systems that guide behavior based on immediate gains and losses. Insufficient top-down control has been proposed as a transdiagnostic mechanism underlying addictive behaviors, allowing reward-driven impulses to dominate behavior despite long-term negative consequences^1,2^. Within this framework, impulsive decision-making processes such as delay discounting, probability discounting, and loss aversion reflect alterations in value-based decision-making rather than executive control per se. For instance, reduced loss aversion, as conceptualized in prospect theory^3,4^, implies diminished sensitivity to potential losses relative to gains, which may facilitate continued gambling despite accumulating negative outcomes. Importantly, these altered processes may be especially relevant for the persistence and escalation of gambling-related symptoms over time. In line with these theoretical assumptions, individuals with GD show various impairments in EFs, e.g. in cognitive flexibility^5–7^, working memory^8^ and most prominently in response inhibition^9–11^. They also show impulsive decision-making for instance in delay and probability discounting^12,13^ and reduced loss aversion^14^. These impairments are not only present in individuals with GD, but also in continuous presentations of different severity below the clinical threshold of a GD diagnosis^12,13^. To refer to individuals on the entire continuum of disordered gambling behavior both below and above the clinical threshold of a diagnosis, we will subsequently use the term disordered gambling. Impairments in EFs and decision-making are discussed as neurocognitive mechanisms implied in GD etiology and paradigmatic of an insufficient top-down regulation of behavior by long term goals^1^ potentially resulting in adverse social, financial, and health consequences. This is especially interesting since GD, as a behavioral addiction, is not associated with substance induced neurological changes, offering the potential to disentangle whether these impaired processes predispose individuals to addictive behaviors or might be a consequence of them^7^. In the following, we will present the literature individually for EFs and impulsive decision-making.

### Executive functions in gambling disorder

Current research suggests individuals with GD show preserved general EFs, but deficits in specific processes like response inhibition, cognitive flexibility, and working memory^15^. There are some inconsistent findings in cognitive flexibility^16–19^, working memory^8,19,20^ and also, even if the most consistent of these aspects, in response inhibition^9,21^. Nevertheless, deficits in EFs like the ones mentioned above are largely accepted as core mechanisms underlying GD. The inconsistencies could in part be due to the multitude of used measures (concerning both tests and respective outcomes), which partly also measure different aspects of respective EFs (esp. in working memory^8^ or tap a number of complex executive processes in one task (e.g. Intra-extradimensional set shift as an alternative measure for cognitive flexibility also requires visual processing^7^. Furthermore, most studies compare individuals with GD with healthy controls^9,17,18^ and a lot of them use treatment-seeking samples^8,9^, thereby neglecting potential different nuances of the continuous association between disordered gambling and EFs. Studies with continuous designs suggest a linear association, where more severely affected individuals exhibit more strongly impaired EFs^5,6,8,22^. Recent studies also favor a dimensional perspective on disordered gambling to reflect its complex, multifactorial nature instead of the oversimplified distinct characterization a binary diagnosis implies^23^.

Moreover, most studies examining EFs in GD are cross-sectional and consequently do not allow any inferences on the course of GD symptoms. However, several studies found deficits in EFs to predict treatment outcomes like drop-out^24^, low compliance and relapse^25,26^, implying the importance of these deficits over the course of GD. There is however a shortage of longitudinal studies exploring the association of deficits in EFs and GD symptoms over time. Evidence from longitudinal studies on substance-related and addictive behaviors / disorders further stresses the importance of deficits in EFs over time as they have been shown to predict the development of addictive behavior^27^.

### Impulsive decision-making in gambling disorder

When it comes to impulsive decision-making, it is important to take into account whether the potential choices are delayed or uncertain^28^ and whether they involve a gain or a loss^29^. There is evidence that individuals with disordered gambling or GD show steeper delay discounting^12,30,31^, more shallow probability discounting, i.e. overvaluing low probability gains and/or undervaluing high probability losses^13^ and less loss aversion^14,32^. Like with deficits in EFs, there are some mixed findings concerning loss aversion^33,34^, but the meta-analyses mentioned above found robust associations of both delay and probability discounting with GD. These effects seem to be stronger when comparing individuals with a GD diagnosis to healthy controls as opposed to continuous designs^12,13^.

Studies on impulsive decision-making are rather heterogeneous, differing in the magnitude of the rewards in question, the length of the delays, the number of choices offered, hypothetical and realistic scenarios and the tasks used to measure these processes^13,31^. Like with EFs, there is a lack of longitudinal studies examining the role of impulsive decision-making in disordered gambling over time. In a recent study of our lab, exploring the same facets of impulsive decision-making mentioned above, lower probability discounting for losses and lower loss aversion predicted symptom increase in addictive behaviors, including gambling^35^. Moreover, more shallow probability discounting was found to predict treatment efficacy in GD^36^ and delayed discounting predicted treatment outcomes in other addictive disorders^12^, thus underlining the importance of these processes for the course, and especially the treatment, of GD.

### Summary and present study

Both, deficits in EFs and impulsive decision-making have been proposed as transdiagnostic processes in addiction^1,2,7,11,12,31^, further emphasizing their importance. This is why, for a better understanding of disordered gamblers and ultimately appropriate treatment options, delineating the temporal role of these distinct processes over the course of disordered gambling is crucial. This need is even bigger, considering the heterogeneous evidence and missing longitudinal studies in the field. There is a further need for studies with continuous approaches, taking the different nuances within the spectrum of disordered gambling into account. Further, there is a need to examine samples of online sports bettors, which - as a popular example of online gambling - gain more and more relevance^37–39^.

Guided by dual-process models of addiction and value-based decision-making frameworks, the present study aimed to examine whether each pre-specified executive control process (response inhibition, cognitive flexibility, working memory) and each alteration in valuation-based decision-making (delay discounting, probability discounting for gains, probability discounting for losses, loss aversion) is differentially associated with gambling disorder symptoms and their change over time in online sports bettors. Sports betting represents a form of gambling that is characterized by repeated decisions under uncertainty, delayed and probabilistic outcomes, and a strong illusion of skill-based control. These features place particular demands on both EFs as well as on value-based decision-making processes, including probability weighting and sensitivity to potential losses. Unlike purely chance-based gambling forms, sports betting requires continuous evaluation of changing information (e.g., odds, session dynamics) and repeated adjustment of decisions, which makes it a theoretically relevant context for studying the interplay between executive function processes and decision-making mechanisms in relation to gambling-related symptoms.

As a first step, we aimed to validate cross-sectional findings concerning deficits in executive function performance and impulsive decision-making in online sports bettors. Concerning our main aim, we explored these processes in association to the change in GD symptoms over time. The present analyses are based on GD symptoms assessed during an initial online survey and a subsequent in-person assessment approximately one year later. We expected the number of GD criteria fulfilled at the in-person assessment, as well as the change in GD criteria from the initial online survey to the in-person assessment, to be negatively associated with performance in executive function tasks. This means that people with an increase (i.e. an aggravation) of GD symptoms would perform worse on executive function tasks. We also expected the number of GD criteria fulfilled at the in-person assessment, as well as the change in GD criteria from the initial online survey to the in-person assessment, to be associated with more impulsive decision-making. That is to say we expected a positive association with delay discounting and negative associations with probability discounting and loss aversion. Cross-sectional hypotheses correspond to H46-52; hypotheses concerning change in GD symptoms to H81-87 of the preregistration of the in-person study (https://osf.io/g3nfv). Importantly, each cognitive process was examined as a theoretically distinct construct, and no overarching inference about “cognitive functioning” as a unified domain was intended.

## Methods

This study is part of the RIGAB project, which includes an initial online survey, a one-year follow-up online survey, and a nested in-person study with a subsample (see https://osf.io/k6c23/ and the study protocol for more details^40^. The analyses presented here are based on data from the initial online survey and the in-person subsample. Hypotheses and analysis plans for each study component were preregistered separately on the Open Science Framework (initial online survey https://osf.io/jbfhe, in-person study https://osf.io/g3nfv)^41,42^. Prior to data collection, we also received aggregated anonymous player tracking data as part of the RIGAB project, though these data were not used in the current analyses.

### Participants and recruitment

#### Sample size rationale

Sample size considerations were based on an a priori power analysis conducted for the preregistered in-person study^42^. Using G*Power 3.1^43^, we calculated the required sample size for multiple linear regression assuming a medium effect size (R² = 0.15), a power of 1 − β = 0.80, and a significance level of α = 0.05, with four covariates. This calculation was informed by the effect sizes reported in previous studies on impulsive decision-making that applied the same paradigms to individuals with addictive behaviours^35^. This yielded a target sample size of *n* = 80 (40 participants per group). Due to substantial difficulties in recruiting participants for in-person testing, this target sample size could not be achieved. Therefore, a secondary power calculation using the same parameters but assuming a single main predictor was conducted to estimate the minimum required sample size. This analysis indicated a minimum sample size of *n* = 50. The final in-person sample (*n* = 54) slightly exceeded this minimum threshold.

#### Recruitment

Participant recruitment was conducted in collaboration with Tipico, an international gambling provider, which provided us with anonymized player tracking data for their entire German customer base. Inclusion criteria for our participants were as follows: aged 18 to 55 years, residing near one of the study locations in Germany (Dresden, Leipzig, Chemnitz, Munich, Berlin, Hamburg, Frankfurt am Main, Düsseldorf), having logged into their account within the two months preceding the study, and having held their account for at least six months. The last two locations were added after initial recruitment began, due to low response rates in the other study locations^44,45^. To facilitate the inclusion of individuals at risk for GD, we used a classification system based on a machine learning algorithm developed by the provider as part of its responsible gambling strategy. This artificial intelligence (AI) algorithm, which continuously evaluates behavior and communication data, categorized customers into groups with or without indicators of risky gambling. Tipico reports that the algorithm performs well in identifying gambling-related issues^46^. The classification was used solely for sampling purposes. After classification, participants were randomly selected for invitation to the study. Due to data protection constraints, the provider was responsible for contacting selected participants.

#### Participants

**Fig. 1:**
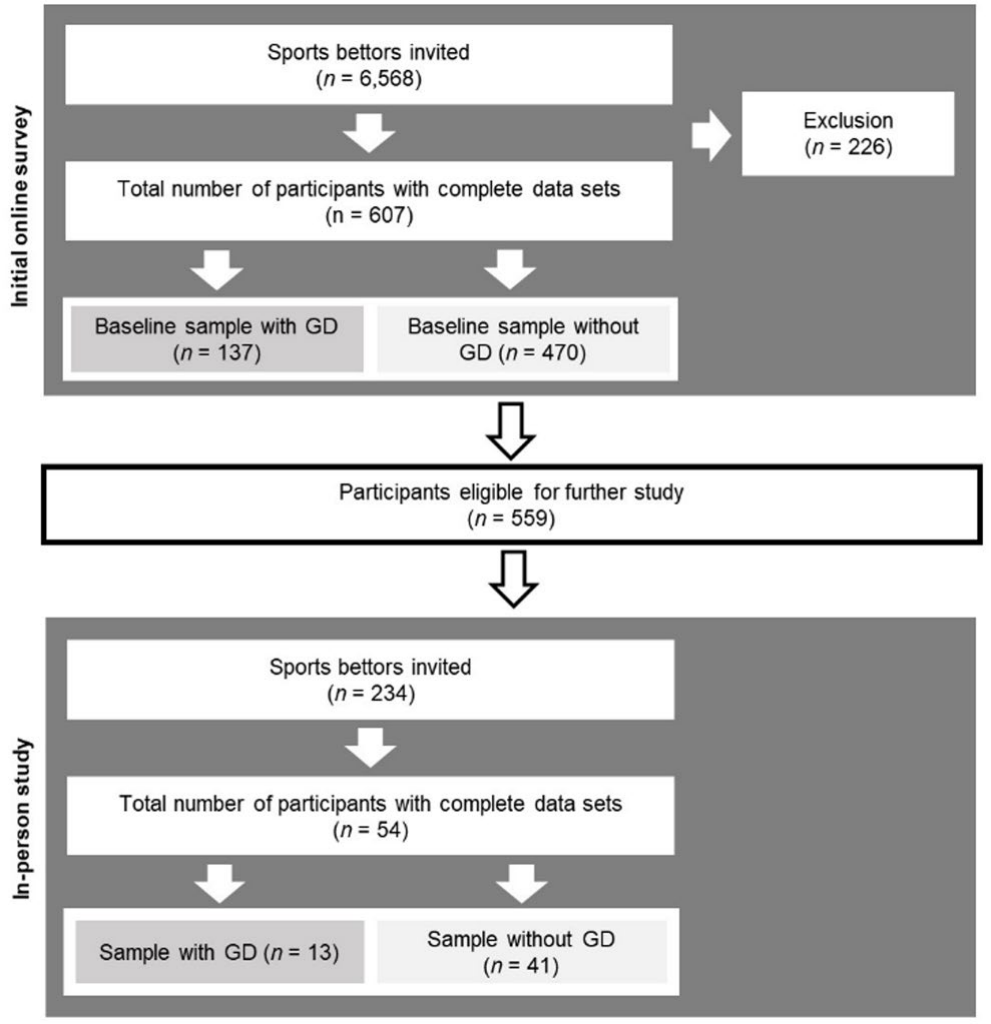
Flowchart of the participants of the RIGAB study (without the online follow-up survey, which was not included in this paper). GD = gambling disorder.

Originally, the in-person subsample was designed to include two distinct groups: participants with and without GD (please see the preregistration of the initial online survey for the original power analysis https://osf.io/jbfhe). However, due to low participation among those with GD, the recruitment strategy was adapted. Participants were instead selected across the full spectrum of GD criteria, starting with individuals who met 3–9 criteria and gradually including those with fewer criteria. Recruitment within each symptom level continued until all eligible individuals had been contacted. Because of anticipated low turnout and logistical barriers (e.g., failed contact attempts), participants were not randomized. Only individuals from Dresden, Leipzig, Chemnitz, and Berlin were invited to the in-person study. A flowchart of the participants of the RIGAB study can be found in Fig. 1.

### Compliance with ethical standards

All study activities adhered to the principles outlined in the Declaration of Helsinki. The study protocol was approved by the Institutional Review Board (IRB00001473) at TUD Dresden University of Technology (reference number SR-EK-190032021). Before beginning the initial online survey, participants were fully informed about the study and provided written informed consent.

### Measures

#### Gambling disorder

GD criteria were assessed using an internally translated German version of the Diagnostic and Statistical Manual of Mental Disorders, fifths edition (DSM-5)[^47^ ] developed by Stichfield, referring to the last 12 months^48^. The initial online assessment measured symptoms using the German version of the Stinchfield DSM-5 GD criteria. During the in-person assessment, the DSM-5 GD criteria were evaluated using a self-developed GD section of the German WHO Composite International Diagnostic Interview (DIA-X, M-CIDI)^49^, which was based on the same German Stinchfield DSM-5 items. Therefore, both instruments assess the same DSM-5 criteria using identical wording. However, the item structure differs: the Stinchfield online questionnaire uses one item per criterion, whereas the M-CIDI uses one item for three criteria and two items for six criteria, with criterion-specific endorsement rules. The DSM-5 criteria have demonstrated satisfactory reliability, validity, and classification accuracy^50^. While various DSM-5-based screenings have been employed in several epidemiological studies in Germany^51^, there are no German validation studies. Therefore, we computed Cronbach’s alpha based on our dataset and found the internal consistency to be 0.84 during the initial assessment and 0.87 during the subsequent online follow-up. Participants responded to questions covering all nine DSM-5 diagnostic criteria for GD using a binary response format (yes/no). To calculate the change in GD criteria, we used the difference between the number of fulfilled criteria from the initial online survey to the in-person study. Positive values indicate an increase in the number of DSM-5 criteria and thus an aggravation of symptoms, negative values indicate a decrease in the number of DSM-5 criteria and therefore an improvement in symptoms. Zero indicates no change between the two times of measurement.

#### Executive functions

The following three tasks were used to assess individual differences in EFs: GoNoGo (response inhibition), Number-Letter (cognitive flexibility) and Two-back (working memory). For a detailed description of the tasks and the respective outcomes see Table 1. For analyses we used inverse efficiency scores (IES)[ ^52^ ], where error rates (ERs) and reaction times (RTs) for each task are combined. IES were calculated using the following procedures already established in our lab^53,54^: to account for individual differences in the balance of the speed-accuracy trade-off^55^ the mean RT of correct responses was divided by the proportion of correct responses (RT/[1 –ER]).

ERs included only wrong-key errors. RTs for error trials and trials immediately following wrong-key errors were excluded. RTs below 100 ms and RTs deviating from the median by more than 3.32 median absolute deviations were also excluded^56^.

Please note that individuals with lower EFs are assumed to display higher IES.

**Table 1:** Executive functions tasks and respective outcomes. (adapted from Wolff et al. 2021).

Task (Executive functions)	Description	Outcome
GoNoGo (response inhibition)	During each trial, a fixation cross was presented for 750 ms, followed by the display of two dots arranged either vertically or horizontally for 500 ms. Participants were directed to press the response key for “Go” trials (vertically arranged dots), while inhibiting the response on “No-Go” trials (horizontally arranged dots). There were 280 “Go” trials and 40 “No-Go” trials.	IES using “go” RT and “no-go” ER
Number-Letter (cognitive flexibility)	Throughout the task, the screen was partitioned into four quadrants by a horizontal and a vertical line. Each trial presented a digit-letter pair (e.g., 2a or g9) for 3,000 ms in one quadrant. Digits were categorized as even or odd, while letters were classified as vowels or consonants. Participants were instructed to utilize two response keys to indicate whether the digit was even or odd (when the stimulus was displayed above the horizontal line), or to determine whether the letter was a vowel or a consonant (when the stimulus was below the horizontal line). “Switch” trials alternated with “no-switch” trials as the stimulus location was rotated in a clock-wise direction from trial to trial. In total, there were 128 trials.	IES difference between “switch” and “no-switch” trials
Two-back (working memory)	During the task, 8 circles positioned around the centre of the screen were displayed. Initially, for the first 1,500 ms of each trial, all circles remained empty. Subsequently, one of the circles was filled for 500 ms, creating a flashing impression. Participants were directed to press the right key when the flashing circle matched the one that had flashed two trials earlier (“yes” trials), and to press the left key otherwise (“no” trials). There were 40 “yes” trials and 120 “no” trials.	IES across all trials

MS = milliseconds, IES = inverse efficiency scores, RT = response time, ER = error rate. Depending on the number of response keys in a task, response keys were “space” (one key) or “Y” and “M” (two keys). Note that “Y” and “Z” keys are transposed on German compared to English-layout keyboards.

#### Impulsive decision-making

To assess impulsive decision-making, we employed four tasks developed by Pooseh et al. (MATLAB scripts accessible from https://github.com/spooseh/VBDM).^57^ These tasks encompassed: (1) a delay discounting task with delays of 3, 7, 14, 31, 61, 180, and 365 days between choice options; probability discounting tasks for (2) gains and (3) losses, involving five probability values (2/3, 1/2, 1/3, 1/4, and 1/5). Each task comprised 30 trials, with monetary gains/losses ranging from 0.30 to 10 €. In the (4) mixed gambles task, participants began with 10 € and played 40 trials with gains ranging from 1 to 40 € and losses from 5 to 20 €. Scenarios were hypothetical, there was no extra compensation for these tasks.

In all four tasks, participants were required to choose between two given options, which were simultaneously presented on a computer screen, using the Psychophysics Toolbox^58^ in MATLAB R2010a^59^. A Bayesian adaptive algorithm was implemented, updating parameter estimation after each trial, and utilizing it for calculating options in the subsequent trial. This approach focused on providing the most informative offers near the individual’s indifference point (the point of indifference between two choice alternatives), enabling efficient inference of decision-making parameters without post-hoc estimations.

For the delay and probability discounting tasks, a hyperbolic value function^60^ was utilized, reflecting that subjective values of delayed (or probabilistic) rewards decrease hyperbolically according to the discounting rate (*k*). In the mixed gambles task, a simple linear function was used, in which loss aversion (*λ*) represents the relative weighting of losses to gains in the participant’s decision^61^. Individuals with higher impulsive decision-making are assumed to display higher *k* values in the delay discounting task, lower *k* values in probability discounting tasks, and lower *λ* values in the mixed gambles task.

### Procedure

The RIGAB (“Characteristics and prediction of RIsky GAmbling Behavior”) study is a longitudinal research project composed of three phases^44,62^. The initial online survey was conducted at baseline. Approximately 12 months later, participants who had consented to being followed up were invited to complete a second online survey. In a nested subsample, an in-person assessment took place on average 443 days (SD = 116) after the initial online survey, with variability in the interval due to logistical factors. Although the broader RIGAB study includes a 12-month online follow-up, the present analyses focus exclusively on data from the initial online survey and the in-person subsample (Fig. 1).

**Fig. 2:**
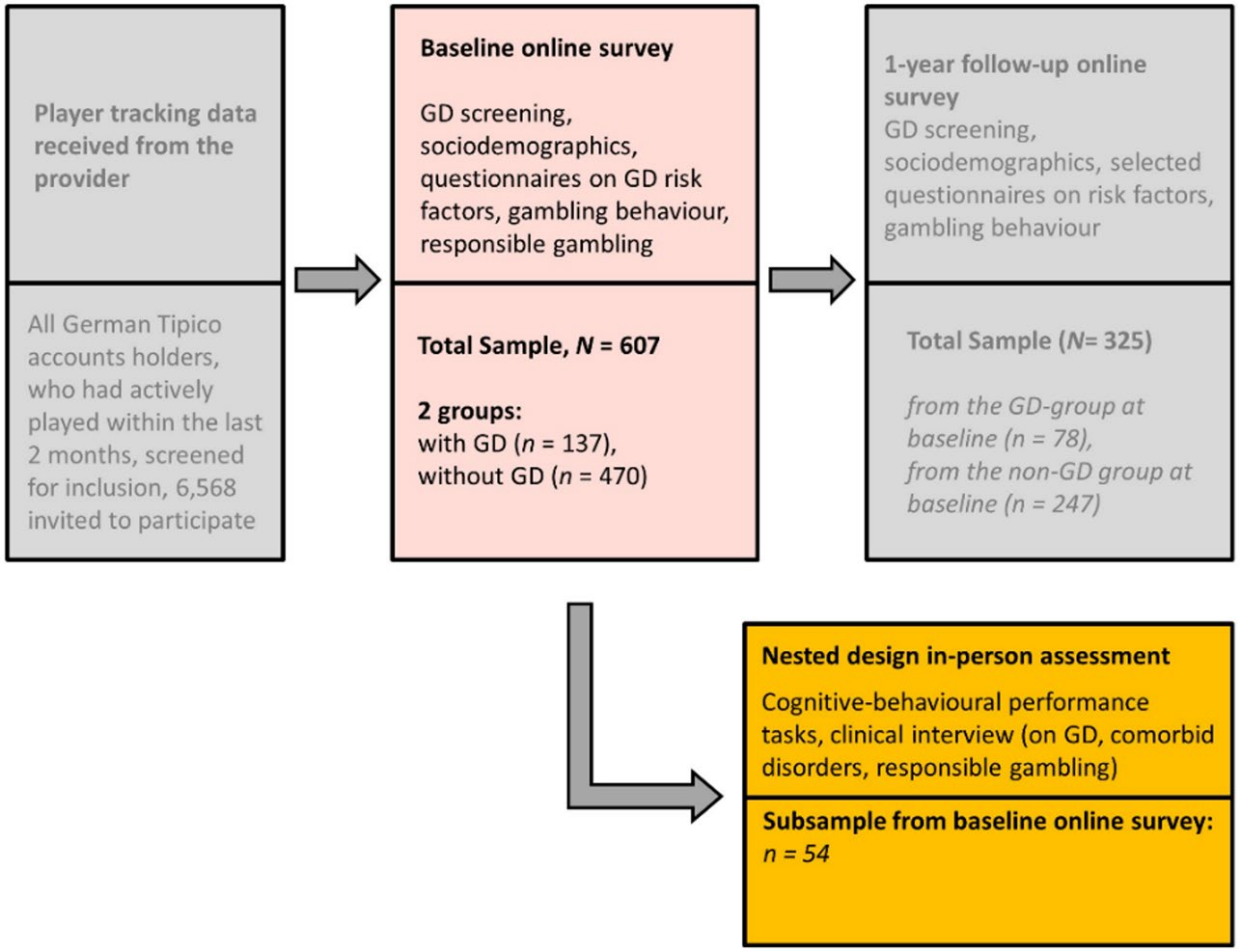
Study design of the RIGAB study^63^. Grey boxes: These parts of the study were not analysed in this paper. GD = gambling disorder assessed with DSM-5 Stinchfield screening questionnaire [^48^].

#### Initial online survey

Data collection for both online surveys was conducted using REDCap^64,65^, a secure, web-based platform hosted by TU Dresden. After providing informed consent, participants completed the assigned questionnaires. A full list of the instruments used is available at https://osf.io/ac8gj^66^. The questionnaires covered sociodemographic, personality, and gambling-related characteristics. During the assessment, participants provided information on the following covariates: gender (female, male or other), age, and level of education. Data on race and ethnicity were not collected. The survey sequence was standardized for all participants. The initial online survey took approximately 30 min to complete. Participants received a €30 Amazon voucher as compensation. After finishing the initial survey, individuals could choose to share their contact information for possible inclusion in future study phases.

#### In-person study

The in-person study took place at TUD Dresden University of Technology and the collaborating Psychologische Hochschule Berlin. During this session, participants undertook a battery of executive function and impulsive decision-making tasks (described above), a structured clinical interview using the Composite International Diagnostic Interview (DIA-X / M-CIDI)^67^, and an interview regarding specific responsible gambling strategies via REDCap. Interview data are not included in the present analysis. The full procedure lasted around two hours and was administered in a fixed order by trained psychologists. Participants were compensated with a €50 Amazon voucher. Those traveling from Leipzig and Chemnitz also received reimbursement for transportation expenses.

### Statistical analysis

All statistical analyses were performed using STATA version 14.2 software^68^. Initially, we sought to examine the cross-sectional relationships between the number of GD criteria fulfilled, according to the DSM-5, and each pre-specified parameter of EFs and impulsive decision-making. To do so, we ran separate multiple linear regression models, using the IES from each executive function task and the k/λ parameters from each impulsive decision-making task as dependent variables and the number of GD criteria met as the independent variable.

Next, we investigated whether changes in GD criteria between the initial online survey and the subsequent in-person assessment (independent variable) were linked to the respective executive function and impulsive decision-making parameters (dependent variables). Change in gambling-related symptoms was operationalized as the difference between symptom counts at baseline and follow-up. Given variability in the follow-up interval across participants, the number of days between assessments was included as a covariate in all longitudinal regression models. As mentioned above we are using IES, where higher IES values indicate impaired EFs. This means that due to the nature of the outcome we expect a positive association of (change in) GD criteria and IES. For example, someone with an increase in GD criteria from the initial online survey to the in-person study is expected to perform worse on executive function tasks, which means to have a higher IES.

Our preregistered hypotheses concerning this were as follows:

We expect the number of GD criteria at the in-person study / the change in GD criteria from the initial online survey to the in-person study to be ….

… positively associated with IES at the GoNoGo task (H46, H81), the Number-Letter task (H47, H82) and the 2-back task (H48, H83).

… positively associated with delay discounting (H49, H84) and negatively associated with probability discounting for gains (H50, H85), losses (H51, H86) and with loss aversion (H52, H87).

Each regression model corresponded to a pre-registered, theoretically distinct hypothesis referring to a specific cognitive parameter. No composite cognitive score or overarching domain-level inference was tested and no joint hypothesis across cognitive domains were evaluated. Covariates included in all analyses were age, gender, educational level, and the age of the participant’s betting account, used as a proxy measure for exposure to online gambling, based on prior literature^69–71^. Additional covariates for the analyses examining criteria changes comprised the number of days elapsed between assessments and the baseline number of GD criteria, to account for regression to the mean effects.

Predictor variables were entered in their original metric and were not standardized. To ensure robustness of the regression analyses, models were estimated using robust standard errors. The robust regression models yielded no meaningful differences, so only the results from the multiple linear regressions are reported. Although hypotheses were directional and preregistered, all p-values reported are based on two-tailed tests, reflecting a conservative testing approach. Given that each model tested a pre-registered and theoretically distinct hypothesis pertaining to a specific cognitive parameter, no correction for multiple testing was applied in the primary analyses. However, as a sensitivity analysis, we additionally controlled the false discovery rate across the seven cognitive outcomes using the Benjamini–Hochberg procedure within each analysis set (cross-sectional and longitudinal models).

To examine whether comorbid substance use disorders influenced the observed associations, a further sensitivity analyses were conducted excluding participants with comorbid alcohol or tobacco use disorders (*n* = 13). All regression models were re-estimated in this reduced sample using the same model specifications as in the primary analyses. These sensitivity analyses were not preregistered and are reported for robustness purposes.

## Results

Descriptive characteristics of the sample from the in-person study can be found in Table 2. Participants were predominantly male (96%) with an average age of 33 years. The majority had a high level of education (*n* = 38 (70%)) and on average the age of their betting account was 4.70 (SD = 2.86) years. The sample was largely homogeneous with respect to cultural background. All participants reported German citizenship (*n* = 54, 100%), and the majority were born in Germany (*n* = 53, 98%) and indicated German as their native language (*n* = 50, 93%). Two participants (4%) reported an additional second citizenship (Iran and Finland). Parental citizenship showed a similar pattern. In most cases, both parents held German citizenship (*n* = 46, 85%). In a small number of cases, one parent held German citizenship while the other parent had a different citizenship, including Turkey (*n* = 1), Poland (*n* = 1), Finland (*n* = 1), and Russia (*n* = 1). Additionally, there were isolated cases in which both parents held the same non-German citizenship (Iran, *n* = 1; Vietnam, *n* = 1). Overall, these data indicate that the sample was predominantly composed of participants with German citizenship and largely German parental background, with only limited representation of more diverse migration-related characteristics.

At the initial online survey, the average number of fulfilled DSM-5 criteria for GD was 2.2 (2.18), at the in-person study it was 2.78 (2.3). The number of fulfilled GD criteria changed between − 4 and + 6 between the initial online assessment and the in-person assessment (see Table S3 in the supplemental material). Eighteen participants (33.3%) showed no change in the number of criteria fulfilled. Eleven participants (20.4%) showed a decrease, while 25 (46.3%) showed an increase. Overall, 63.0% of participants exhibited changes of between − 1 and + 1 criteria.

For descriptive data on the initial and the follow-up online survey respectively, please see our other publications^23,45^. Descriptive statistics of the respective executive function and impulsive decision-making tasks can be found in Table 3. The correlations between the cognitive outcome parameters were generally small (see Table S4 in the supplementary material), which indicates that the measures were largely independent of each other.

**Table 2:** Descriptive characterization of the in-person sample.

	M (SD) / Absolute frequencies (%)	Range
Age	33 (10.04)	20–55
Gender (male)	*n* = 52 (96%)	
Education		
Middle	*n* = 16 (30%)	
High	*n* = 38 (70%)	
Sum of DSM-5 Stinchfield criteria at initial online survey	2.22 (2.18)	0–9
Sum of DSM-5 Stinchfield criteria at in-person study	2.78 (2.30)	0–8
Number of days between initial online survey and in-person study	443.41 (116.31)	234–699
Age of betting account	4.70 years (2.86)	0.93–12.81
German as a native language	*n* = 50 (93%)	
German citizenship	*n* = 54 (100%)	
Most common family status	*n* = 42 single (78%)	
Most common employment status	*n* = 32 fully employed (59%)	

*N*_*total sample*_ = 54. *M* = Mean, *SD* = Standard deviation.

**Table 3:** Descriptive statistics and reliabilities of the executive function and impulsive decision-making tasks.

Outcome	M	SD
Executive functions (IES)		
GoNoGo	351.71^a^	59.74
Number-Letter	382.11^b^	201.92
Two-back	555.98^c^	148.47
Impulsive decision-making		
Delay discounting (log *k*)	-5.21	2.89
Probability discounting for losses (log *k*)	− 0.32	2.58
Probability discounting for wins (log *k*)	0.01	0.84
Loss aversion (log *λ*)	0.24	0.54

If not otherwise indicated *N* = 54. *M* = Mean, *SD* = Standard Deviation, IES = Inverse Efficiency Scores.

^a^ *n* = 52, 2 persons had to be excluded due to exclusion procedures explained above.

^b^ *n* = 47, 2 persons could not complete this task due to technical difficulties, 5 persons had to be excluded due to exclusion procedures explained above.

^c^ *n* = 51, 3 persons had to be excluded due to exclusion procedures explained above.

### Cross-sectional associations of executive functions, decision-making and gambling disorder criteria

We found no evidence of a significant association between the number of fulfilled GD criteria at the in-person study and performance in executive function tasks, except for the two-back task (working memory). Participants with more GD criteria showed higher IES on the two-back task (*b* = 23.98, *p* = .027 [2.82, 45.15]). This association did not remain statistically significant after false discovery rate correction (q = 0.096).

Concerning impulsive decision-making we found a significant association between the number of GD criteria at the in-person study and loss aversion; participants with more GD criteria were less loss averse and vice versa (*b* = − 0.09, *p* = .024, [-0.16, − 0.01]). This association did not remain statistically significant after false discovery rate correction (q = 0.096). All other associations were not significant. Results of the multiple regressions for the association of GD criteria at the in-person study and EFs and impulsive decision-making can be found in Table 4.

Sensitivity analyses excluding participants with comorbid alcohol or tobacco use disorders (*n* = 13) revealed that the association between loss aversion and the number of GD criteria remained nominally significant, whereas the association with working memory did not (see Table S4 in the supplement).

**Table 4:** Results of the multiple linear regression analyses for cross-sectional association of executive functions, impulsive decision-making and gambling disorder criteria at the in-person study.

Outcome	Regression coefficient	*p*	q (FDR)	95% CI
Executive functions (IES)				
GoNoGo	− 0.06^a^	0.988	0.989	[– 8.26, 8.14]
Number letter	12.40^b^	0.431	0.754	[– 19.11, 43.91]
Two-back	23.98^c^	0.027	0.096	[2.82, 45.15]
Impulsive decision-making				
Delay discounting (log *k*)	-0.003	0.989	0.989	[– 0.39, 0.39]
Probability discounting for losses (log *k*)	0.19	0.254	0.592	[– 0.14, 0.52]
Probability discounting for wins (log *k*)	− 0.03	0.649	0.909	[– 0.14, 0.09]
Loss aversion (log *λ*)	− 0.09	0.024	0.096	[– 0.16, – 0 0.01]

If not otherwise indicated *N* = 54. CI = 95% Confidence Interval, IES = Inverse Efficiency Scores. All analyses controlled for the influence of age, gender, education and age of account. All *p* values are two-tailed. The *q* values represent false discovery rate (FDR)–adjusted *p* values (Benjamini–Hochberg procedure) across the seven cognitive outcomes. Results are generally reported using two decimal places, with three decimal places used for *p* and *q* values or when rounding to two decimals would result in a value of zero.

^a^ *n* = 52, 2 persons had to be excluded due to exclusion procedures explained above.

^b^ *n* = 47, 2 persons could not complete this task due to technical difficulties, 5 persons had to be excluded due to exclusion procedures explained above.

^c^ *n* = 51, 3 persons had to be excluded due to exclusion procedures explained above.

### Associations of executive functions, decision-making and change in GD criteria over time

In contrast to our hypotheses, only the association between loss aversion and the change in GD criteria from initial online survey to in-person study was significant. Participants with a positive change in GD criteria and thus an aggravation of symptoms were less loss averse and vice versa (*b* = − 0.09, *p* = .045, [-0.17, -0.002]). This association did not remain statistically significant after false discovery rate correction (q = 0.314). All other associations between the change in GD criteria and EFs and impulsive decision-making respectively were not significant. Results of the multiple regressions for the association of change in GD criteria from the initial online survey to the in-person study and EFs and impulsive decision-making can be found in Table 5. Sensitivity analyses that excluded participants with comorbid alcohol or tobacco use disorders (*n* = 13) corroborated the main finding. The longitudinal association between loss aversion and changes in gambling-related symptoms remained nominally significant (see Table S5 in the supplement).

**Table 5:** Results of the multiple linear regression analyses for the association of executive functions, impulsive decision-making and the change in gambling disorder criteria from the initial online survey to the in-person study.

Outcome	Regression coefficient	*p*	q (FDR)	95% CI
Executive functions (IES)				
GoNoGo	− .65^a^	0.895	0.948	[-10.52, 9.21]
Number Letter	-1.22^b^	0.948	0.948	[-38.82, 36.37]
Two-back	16.56^c^	0.173	0.453	[-7.58, 40.69]
Impulsive decision-making				
Delay discounting (log k)	0.16	0.473	0.828	[-0.29, 0.61]
Probability discounting for losses (log k)	0.25	0.194	0.453	[-0.13, 0.64]
Probability discounting for wins (log k)	0.03	0.633	0.886	[-0.10, 0.16]
Loss aversion (log λ)	− 0.09	0.045	0.314	[-0.17, − 0.002]

If not otherwise indicated *N* = 54. CI = 95% Confidence Interval. All analyses controlled for the influence of age, gender, education, age of account, days between initial online survey and in-person study and GD criteria at initial online survey. All *p* values are two-tailed. The *q* values represent false discovery rate (FDR)–adjusted *p* values (Benjamini–Hochberg procedure) across the seven cognitive outcomes. Results are generally reported using two decimal places, with three decimal places used for *p* and *q* values or when rounding to two decimals would result in a value of zero.

^a^ *n* = 53, 1 person had to be excluded due to exclusion procedures explained above.

^b^ *n* = 47, 2 persons could not complete this task due to technical difficulties, 5 persons had to be excluded due to exclusion procedures explained above.

^c^ *n* = 51, 3 persons had to be excluded due to exclusion procedures explained above.

## Discussion

Our main aim for the present study was to explore the association between EFs, decision-making and the change in GD symptoms over the past year in a sample of online sports bettors; thereby adding to the literature on the temporal role of specific deficits in these processes in relation to changes in GD symptoms. Importantly, the present study focuses on online sports bettors with the majority of participants not meeting the diagnostic threshold for GD. Consistent with dimensional models of GD symptoms^73^, disordered gambling is therefore treated as a continuous construct rather than a categorical diagnosis. In the first step, a cross-sectional validation, we found nominal associations indicating that more disordered gambling over the past year was associated with more deficits in working memory and reduced loss aversion, but no evidence for deficits in other executive function or decision-making processes. Furthermore, we found nominal associations between loss aversion and more disordered gambling over the past year, but no evidence for an association with other impulsive decision-making processes. However, these associations were no longer statistically significant after controlling for multiple testing, so they should be interpreted with caution.

For the first time our study explored these associations in relation to the change of GD symptoms over time at an average of 443 days later. Our results showed a nominal association between reduced loss aversion and an increase in GD symptoms over time. This association was not statistically significant after correction for multiple testing, so it should be considered preliminary. No evidence was available that other processes were associated with the change of GD symptoms over time. Thus, while the pattern of results may suggest a potential role for reduced loss aversion in symptom dynamics, the findings do not provide robust statistical support for a specific cognitive mechanism underlying changes in GD symptoms. These new findings tentatively underline the relevance of reduced loss aversion in relation to changes in gambling disorder symptoms over time and further stress the need for longitudinal studies on EF and decision-making processes in individuals with disordered gambling. From a theoretical standpoint, the present findings may point to alterations in value-based decision-making, rather than broad deficits in executive control, as being particularly relevant for changes in gambling-related symptoms over time. However, as none of the observed associations remained significant after correction for multiple testing, any interpretation at the domain level should be treated with caution.

Loss aversion is a core construct of prospect theory and reflects the extent to which potential losses are weighted more strongly than equivalent gains during decision-making^3,4^. Reduced loss aversion implies a diminished sensitivity to potential losses, which may facilitate the continuation of gambling behavior despite repeated negative outcomes. Importantly, this valuation-related mechanism is conceptually distinct from EFs and was examined as a separate, pre-specified hypothesis rather than as part of a global cognitive construct. We will discuss our findings individually for each construct below.

### Executive functions

Contrary to our hypotheses, we did not find evidence for associations between deficits in EFs and GD symptom counts assessed over the past year, neither cross-sectionally nor with change in symptoms over time, with the exception of working memory (cross-sectionally). The cross-sectional association between working memory and GD symptoms was nominal and did not survive correction for multiple testing. This is in line with other studies finding only isolated deficits in EFs^17,19^. Overall, the current findings do not suggest that there is a general pattern of impaired EFs among online sports bettors. The cross-sectional association between working memory performance and disordered gambling did also not persist after excluding participants with comorbidities. This pattern suggests that working memory deficits observed in the full sample may, at least in part, reflect comorbidity-related cognitive effects rather than gambling-related processes per se. Whether these differences in working memory performance precede gambling-related problems, needs to be assessed in future longitudinal studies with adolescents.

The otherwise absent evidence for deficits in EFs might be due to the fact that strategic gambling activities like sports betting demand cognitive processes and individuals preferring these might thus not present any or less deficits in EFs as opposed to non-strategic gamblers^74^. For instance, working memory and mental flexibility are necessary to calculate and compare odds and probable payouts; while declarative memory is needed to apply past knowledge and experiences (e.g. performance track records, absence of key players) to assess the probability of wins^18^. Whether these individuals’ underlying neurobiology supports efficient EFs, fostering a preference for strategic gambling, or if regular use of these EFs during betting has contributed in preserving cognitive processes is unclear^18,75^.

Our findings add to existing inconsistencies concerning the facets of EFs explored here and are in line with a number of studies reporting no significant differences between individuals with disordered gambling and healthy controls^5,8,16–21,75,76^. Within dual-process frameworks, this pattern may indicate relatively preserved top-down control in this sample. However, the absent evidence for lower inhibitory control is contradictory to a meta-analysis reporting robust associations^9^.

Our results might also be partially explained by sample and measurement limitations. We examined a relatively small sample, where the majority of participants displayed a low to modest number of GD symptoms below the cut-off of a diagnosis (76% ≤ 3 symptoms at initial online survey, 65% at in-person study, see Tables S1, S2 in the supplement). The association of deficits in EFs and disordered gambling severity^5,26^ and studies finding differences between individuals with GD and healthy controls but not subclinical levels of disordered gambling^6^ suggest a linear association of GD symptoms and deficits in EFs. Consequently, impairments in EFs in our sample might not have been that severe, which in turn might have made it hard to detect an association. The same might have been the case for the change in GD symptoms over time, where most participants exhibited only a minor change in symptoms (no difference for one third, 63% between − 1 and + 1). This is however in line with studies on the course of GD, suggesting that most individuals ”at-risk” of and with GD move toward a lower (or less intensive) level of gambling behavior over time, while those who gamble recreationally are unlikely to move to a more severe level^77,78^. In combination with the modest sample size and the correction for multiple testing applied in the present study, these characteristics may have further reduced statistical power to detect small effects.

Concerning the measurement limitations, performance variability was relatively high for some executive function tasks. However, this pattern is consistent with previous studies using the same paradigm^27^ and likely reflects task-inherent characteristics. Nevertheless, high variability and task-specific exclusions may have reduced sensitivity to detect associations. Furthermore, IESs were used to combine reaction time and accuracy into a single performance index. This approach assumes a relatively consistent speed–accuracy trade-off across individuals, which may not hold for all participants, particularly in gambling-related populations characterized by heterogeneity in response strategies. As a result, the reliability and sensitivity of the executive function tasks to detect associations with disordered gambling may have been reduced, and null findings for this measure should be interpreted with caution.

In addition, the already mentioned sizeable heterogeneity in almost all aspects of methodology in studies concerned with EFs in gambling^15^, further hampers comparisons across studies. For instance, conclusions based on treatment-seeking populations might skew the picture as they are inferred from individuals with a potentially longer duration and higher severity of GD and assumed associated deficits in EFs^27^. However, the heterogeneity of findings also strengthens the argument that GD is a disorder characterized by substantial heterogeneity, for instance also depicted in efforts to subtype its affected individuals (e.g. pathways model of problem and pathological gambling)^79,80^. Potentially this includes a subtype where only highly affected individuals show deficits in EFs, but mildly affected individuals, who may experience difficulties in other areas due to gambling, do not (cf. considerations concerning quasi- or semi-continuous relationships for delay discounting as proposed)^12^. Conceivably, this could interact with other factors like the preferred form of gambling.

Future studies should explore the association of EFs and the course of disordered gambling with a sufficiently distributed variation of severity in longitudinal designs, ideally with larger sample sizes including strategic and non-strategic gamblers. To facilitate comparisons across studies a comprehensive and unitary network of EFs in disordered gambling is needed.

Nevertheless, assuming online sports bettors do have preserved and robust EFs also means individuals with disordered gambling are well capable of participating in and benefiting from cognitive demanding treatment like Cognitive Behavioral Therapy^18^. However, this implication should be interpreted with caution, given the limitations of the present study mentioned above.

### Impulsive decision-making

Reduced loss aversion showed nominal associations with disordered gambling, cross-sectionally and with the change of disordered gambling over time. However, these associations did not withstand correction for multiple testing and therefore cannot be considered statistically robust. These associations were also observed in additional sensitivity analyses excluding participants with comorbid alcohol or tobacco use disorders, suggesting that this effect is not driven by comorbid substance use. This finding is in line with other cross-sectional studies showing that individuals with GD exhibit less loss aversion than healthy controls^33,34,81^. In a study with adolescents with internet gaming disorder the majority of participants showed reduced loss aversion, but no evidence for impaired response inhibition^82^. As only severely affected individuals also showed impairments in response inhibition the authors concluded that mildly affected individuals mainly differ in their reward-seeking system but not in EFs. They posit reduced loss aversion as an important factor for initiation and development of this behavioral addiction similar to GD, which is linked to underestimating long-term negative outcomes due to the disorder. This might further speak for the heterogeneity of cognitive function in disordered gamblers with specific but not general deficits and consequently also domains entirely unaffected.

Our findings suggest that reduced loss aversion may be one of several cognitive processes associated with worsening GD symptoms over time in sports bettors. Reduced loss aversion may represent a cognitive characteristic linked to the persistence of gambling behavior despite negative consequences, a feature commonly observed in more severe forms of disordered gambling. This association may help to explain individual differences in the extent to which negative outcomes are weighed against potential gains. This could in turn be linked to motivational processes in treatment. Accordingly, interventions that focus on strengthening personally relevant values and goals outside of gambling, for example through motivational interviewing^83^, may be particularly useful in supporting motivation for change. However, as the longitudinal association was not statistically significant after correction for multiple testing, these implications should be interpreted with caution.

Despite the longitudinal nature of the study, only two time points were available, which precludes conclusions about the direction of causality. The present findings therefore suggest a possible association between reduced loss aversion and changes in gambling disorder symptoms, but cannot determine whether reduced loss aversion precedes symptom worsening or emerges as a consequence of increasing gambling-related problems. Furthermore, while the observed associations were statistically significant, they did not withstand correction for multiple testing, and their magnitude was small. They should therefore be interpreted with appropriate caution. Reduced loss aversion is likely to be one of several contributing factors, rather than a strong standalone indicator, of symptom change. Future studies should employ cross-lagged panel designs with larger samples and with three or more measurement occasions to disentangle the temporal and potentially causal relationships between EFs, decision-making processes such as loss aversion, and gambling disorder symptoms.

In contrast to our hypotheses and a vast body of literature^12,13^, we did not find any evidence for associations between delay or probability discounting and disordered gambling, both cross-sectionally and concerning the change in symptoms over time. This might be partly attributable to the already mentioned heterogeneity in studies concerning amongst others measures, samples and sample sizes. Akin to deficits in EFs, the low levels of disordered gambling in our sample might have made it difficult to detect impulsive decision-making, as they might have entailed that putative impairments in these processes are not as severe. Although counter-intuitive as continuous designs supposedly have higher power to detect associations, other studies also found larger effect sizes for studies with categorical designs^12,13^. Furthermore, as is the case with EFs, some authors propose there might be differences between strategic and non-strategic gamblers in decision-making^74,81^, which could explain the difference to other studies.

### Limitations

Several limitations should be considered when interpreting the present findings concerning the design, the measures, and the sample. Concerning the design, change over time was assessed using a simple difference score across two time points with varying follow-up intervals. Although the interval was statistically controlled, individual trajectories were not modeled, so observed effects reflect differences between non-uniform time points. Another limitation is the absence of important contextual control variables, such as economic status or family background, to heighten ecological validity. While the focus of our longitudinal analyses on within-person change scores reduces the influence of stable between-person differences, we cannot rule out the impact of time-varying, unmeasured factors on symptom changes (e.g., acute life events, major wins or losses, temporary substance use, or short-term treatment exposure). Consequently, observed associations between cognitive processes and symptom change should be interpreted as indicative of potential mechanisms.

Concerning the measurement limitations, GD symptoms were assessed using the number of DSM-5 criteria met, which reflects the past 12 months rather than current severity. In contrast, EFs and decision-making were assessed cross-sectionally at the time of the in-person assessment so that the observed associations are correlational and not concurrent or causal. Moreover, DSM-5 symptom counts provide a diagnostic framework rather than a fine-grained measure of severity. More sensitive dimensional instruments such as the Gambling Symptom Assessment Scale (G-SAS)^84^ may be better suited to capture variability in gambling symptom severity, particularly in non-clinical samples. A further limitation concerns the assessment of GD criteria across assessments. Although all measures were based on identical DSM-5 criteria and wording, symptom counts were derived from different instruments and assessment modes (online self-report vs. in-person interview). Differences in item structure between the Stinchfield screening and the M-CIDI may influence symptom endorsement patterns and thus affect the interpretation of symptom change.

Although our executive function and decision-making paradigms are well established and in some cases even said to be advantageous over other measures (e.g. Two-back task as a comparatively holistic assessment of working memory)^8^, the kind of behavioral measures used within this study have been criticized for their lack of ecological validity^18^. This concerns for instance the use of generic instead of complex cues or processes in milliseconds instead of more realistic larger time frames^9^. In the case of impulsive decision-making, this might imply that these measures are not suited to detect or activate modest deficiencies in a population well-accustomed to winning/losing real money^18^.

Our sample mainly consists of individuals with a low number of gambling-related symptoms, which limit the estimation of the association between cognitive deficits and moderate-to-severe gambling disorder. Therefore, the present results should be validated in clinical samples of individuals meeting diagnostic criteria for GD. In addition, the sampling strategy oversampled individuals with elevated gambling-related symptoms, which may have contributed to regression-to-the-mean effects, even though baseline symptom levels were controlled for in the analyses. In addition, participants were recruited via a proprietary risk-classification algorithm provided by a gambling provider. Although this algorithm was used exclusively for sampling and did not inform any study measures or analyses, it might have resulted in a systematic preselection of individuals whose gambling behavior aligns with the operator’s internal risk thresholds. Consequently, the findings may not generalize to all sports bettors or to individuals with gambling-related problems identified through alternative screening or clinical pathways. Furthermore, our sample showed a substantial gender imbalance, which limits the generalizability of the findings. Although gender was included as a covariate in all regression models, the sample size and distribution did not allow for the examination of potential gender-specific associations. Given evidence that risk factors for GD may differ by gender^85^, future studies with more balanced samples are needed to investigate whether the observed associations vary across genders. Our sample also lacked direct measures of race or ethnicity, reflecting common research practice in Germany due to historical, ethical, and methodological considerations. While we used proxy indicators such as citizenship, country of birth, native language, and parental citizenship to describe the sample, these do not capture processes of racialization or allow conclusions regarding differences related to race or ethnicity. Future research should consider context-sensitive approaches to better assess diversity and inequality in German samples. Finally, the modest sample size limits statistical power, particularly for detecting small effects, and may have contributed to null findings for some executive function and decision-making measures.

## Conclusion and future research

To the best of our knowledge our study was the first to explore associations between EFs, decision-making, and changes in gambling disorder symptoms over time in online sports bettors. We observed associations between reduced loss aversion and an exacerbation of disordered gambling symptoms. As this association was not statistically robust after correction for multiple testing, it should not be interpreted as evidence of a reliable predictor. Rather, it should be considered a potential variable of interest for future studies requiring replication. The present findings are based on a sample of individuals who predominantly engaged in sports betting, which represents only one specific form of gambling. Other gambling forms differ in their structural characteristics, cognitive demands, and reinforcement schedules. Future studies should therefore test and compare the present hypotheses across different gambling activities to determine whether the observed associations between executive functions, value-based decision-making, and changes in disordered gambling generalize beyond sports betting or are specific to certain gambling forms. To test these hypotheses, future research should examine large samples with varied severity and ideally incorporate not exclusively treatment-seeking, strategic and non-strategic gamblers and assess long follow-up periods.

Our findings further support that individuals with disordered gambling are characterized by substantial heterogeneity with highly individual presentations, where clustering efforts for instance by preferred gambling type might facilitate a more comprehensive understanding of subgroups. While the present results do not provide statistically robust evidence for specific cognitive mechanisms, they are consistent with the broader notion of heterogeneity in cognitive profiles among individuals with gambling-related problems. As (online) gambling becomes increasingly widespread and easily accessible, comprehending the cognitive characteristics of specific subgroups of gamblers with increasing relevance like online sports bettors remains crucial to the implementation of effective treatment strategies.

## Supplementary Information

Below is the link to the electronic supplementary material.

Supplementary Material 1

## Data Availability

The datasets presented in this article are not publicly available due to their inclusion of sensitive information, such as player tracking data. Since the raw data could potentially allow for the identification of individuals, further anonymization, such as data aggregation, is required before any public release. We are currently preparing the data in accordance with FAIR principles and applying the necessary anonymization procedures. This process is expected to be completed by autumn 2026. Afterward, the anonymized dataset will be made publicly available. Sensitive data will be accessible only in aggregated form or through controlled access within an open repository for qualified research purposes. Until then, data may be made available upon reasonable request to the corresponding author.
