# Supplementary material for: The relationship between executive functions, decision-making, and changes in symptoms of gambling disorder in online sports bettors
Source: Sci Rep. 2026 Apr 12;16:12076. doi: 10.1038/s41598-026-48449-8 (PMC13076668; doi:10.1038/s41598-026-48449-8)
Supplement: Supplementary file 1 — Supplementary Material 1 [file 41598_2026_48449_MOESM1_ESM.docx]

**Supplemental material for:**

**The relationship between executive functions, decision-making, and changes in symptoms of gambling disorder in online sports bettors**

In *Scientific Reports*

**Theresa Wirkus^1^, Robert Czernecka^1^, Gerhard Bühringer^1,2^ & Anja Kräplin^3^***

^1^Faculty of Psychology, Technische Universität Dresden, Germany

^2^IFT Mental Health Solutions, Munich, Germany

^3^Department of Psychiatry and Psychotherapy, Technische Universität Dresden, Germany

*** Correspondence:**Anja Kräplin

[anja.kraeplin@tu-dresden.de](mailto:anja.kraeplin@tu-dresden.de)

ORCID: [0000-0002-1612-3932](https://orcid.org/0000-0002-1612-3932)

# Distribution of fulfilled criteria for gambling disorder at both time-points and the respective change in criteria between measurements

**Table S1.** Distribution of fulfilled criteria for gambling disorder for the subsample of the in-person study at initial online survey.

| Number of fulfilled GD criteria | Frequency | Percent | Cumulative percent |
| --- | --- | --- | --- |
| 0 | 14 | 25.93 | 25.93 |
| 1 | 9 | 16.67 | 42.59 |
| 2 | 14 | 25.93 | 68.52 |
| 3 | 4 | 7.41 | 75.93 |
| 4 | 4 | 7.41 | 83.33 |
| 5 | 5 | 9.26 | 92.59 |
| 7 | 3 | 5.56 | 98.15 |
| 9 | 1 | 1.85 | 100.00 |

*Note.* *N* = 54, GD = gambling disorder, assessed via an internally translated German version of the Stinchfield criteria from the Diagnostic and Statistical Manual of Mental Disorders (DSM-5, 5th edition, American Psychiatric Association (APA), 2013), focusing on the last 12 months (modified from Buchner et al., 2009).

**Table S2.** Distribution of fulfilled criteria for gambling disorder at in-person study.

| Number of fulfilled GD criteria | Frequency | Percent | Cumulative percent |
| --- | --- | --- | --- |
| 0 | 9 | 16.67 | 16.67 |
| 1 | 11 | 20.37 | 37.04 |
| 2 | 8 | 14.81 | 51.85 |
| 3 | 7 | 12.96 | 64.81 |
| 4 | 8 | 14.81 | 79.63 |
| 5 | 4 | 7.41 | 87.04 |
| 6 | 2 | 3.70 | 90.74 |
| 7 | 2 | 3.70 | 94.44 |
| 8 | 3 | 5.56 | 100.00 |

*Note.* *N* = 54, GD = gambling disorder, assessed as part of an adapted version of the German WHO Composite International Diagnostic Interview (DIA-X / M-CIDI, Wittchen and Pfister, 1997) at the in-person study.

**Table S3.** Distribution of change in fulfilled criteria for gambling disorder between initial online survey to in-person study.

| Change in number of fulfilled GD criteria | Frequency | Percent | Cumulative percent |
| --- | --- | --- | --- |
| -4 | 3 | 5.56 | 5.56 |
| -3 | 1 | 1.85 | 7.41 |
| -2 | 1 | 1.85 | 9.26 |
| -1 | 6 | 11.11 | 20.37 |
| 0 | 18 | 33.33 | 53.70 |
| 1 | 10 | 18.52 | 72.22 |
| 2 | 8 | 14.81 | 87.04 |
| 3 | 4 | 7.41 | 94.44 |
| 4 | 1 | 1.85 | 96.30 |
| 5 | 1 | 1.85 | 98.15 |
| 6 | 1 | 1.85 | 100.00 |

*Note.* *N* = 54, GD = gambling disorder, assessed via an internally translated German version of the Stinchfield criteria from the Diagnostic and Statistical Manual of Mental Disorders (DSM-5, 5th edition, American Psychiatric Association (APA), 2013), focusing on the last 12 months (modified from Buchner et al., 2009) at the initial online survey and as part of an adapted version of the German WHO Composite International Diagnostic Interview (DIA-X / M-CIDI, Wittchen and Pfister, 1997) at the in-person study.

**Table S4. Spearman rank correlations between executive function and impulsive decision-making parameters**

| **Variable** | **1** | **2** | **3** | **4** | **5** | **6** | **7** |
| --- | --- | --- | --- | --- | --- | --- | --- |
| 1. Delay discounting (log k) | — |  |  |  |  |  |  |
| 2. Loss aversion (log λ) | .26 | — |  |  |  |  |  |
| 3. Probability discounting losses (log k) | .01 | −.15 | — |  |  |  |  |
| 4. Probability discounting wins (log k) | .21 | .03 | .11 | — |  |  |  |
| 5. GoNoGo (IES) | −.13 | −.20 | .26 | .11 | — |  |  |
| 6. Number-Letter (IES) | −.02 | −.19 | .15 | −.17 | .05 | — |  |
| 7. Two-back (IES) | −.13 | −.25 | −.07 | .18 | .23 | .09 | — |

*Note.* N = 43. Values represent Spearman’s rank correlation coefficients (ρ). IES = Inverse Efficiency Score.

**Table S5.** Results of the multiple linear regression analyses for cross-sectional association of executive functions, impulsive decision-making and gambling disorder criteria at the in-person study without *n* = 13 participants with comorbid tobacco use or alcohol use disorder.

| Outcome | Regression coefficient | *p* | 95% CI |
| --- | --- | --- | --- |
| Executive functions (IES) |  |  |  |
| GoNoGo | .40^a^ | .923 | [-7.93, 8.73] |
| Number Letter | 9.05^b^ | .626 | [-28.62, 46.73] |
| Two-back | 13.67^c^ | .192 | [-7.21, 34.54] |
| Impulsive decision-making |  |  |  |
| Delay discounting (log *k*) | -.05 | .850 | [-.53, .44] |
| Probability discounting for losses (log *k*) | .17 | .339 | [-.19, .53] |
| Probability discounting for wins (log *k*) | -.05 | .495 | [-.19, .09] |
| Loss aversion (log *λ*) | -.12 | .005 | [-.20, -.04] |

*Note.* If not otherwise indicated *N* = 41. *SD* = Standard Deviation, CI = 95% Confidence Interval, IES = Inverse Efficiency Scores. All analyses controlled for the influence of age, gender, education and age of account. All *p* values are two-tailed. Results are generally reported using two decimal places, with three decimal places used for *p* values or when rounding to two decimals would result in a value of zero.

^a^ *n* = 40, 1 person had to be excluded due to exclusion procedures explained above

^b^ *n* = 35, 1 person could not complete this task due to technical difficulties, 5 persons had to be excluded due to exclusion procedures explained above

^c^ *n* = 40, 1 person had to be excluded due to exclusion procedures explained above

**Table S6.** Results of the multiple linear regression analyses for the association of executive functions, impulsive decision-making and the change in gambling disorder criteria from the initial online survey to the in-person study without *n* = 13 participants with comorbid tobacco use or alcohol use disorder.

| Outcome | Regression coefficient | *p* | 95% CI |
| --- | --- | --- | --- |
| Executive functions (IES) |  |  |  |
| GoNoGo | -1.29^a^ | .795 | [-11.36, 8.78] |
| Number Letter | -3.96^b^ | .862 | [-50.21, 42.29] |
| Two-back | 10.35^c^ | .384 | [-13.53, 34.22] |
| Impulsive decision-making |  |  |  |
| Delay discounting (log k) | .16 | .561 | [-.39, .70] |
| Probability discounting for losses (log k) | .30 | .144 | [-.11, .71] |
| Probability discounting for wins (log k) | .01 | .903 | [-.15, .17] |
| Loss aversion (log λ) | -.10 | .032 | [-.19, -.01] |

*Note.* If not otherwise indicated *N* = 41. *SD* = Standard Deviation, CI = 95% Confidence Interval. All analyses controlled for the influence of age, gender, education, age of account, days between initial online survey and in-person study and GD criteria at initial online survey. All *p* values are two-tailed. Results are generally reported using two decimal places, with three decimal places used for *p* values or when rounding to two decimals would result in a value of zero.

^a^ *n* = 40, 1 person had to be excluded due to exclusion procedures explained above

^b^ *n* = 35, 1 person could not complete this task due to technical difficulties, 5 persons had to be excluded due to exclusion procedures explained above

^c^ *n* = 40, 1 person had to be excluded due to exclusion procedures explained above

**Please see the original manuscript for a full list of references.**
